# Supplementary material for: Distinct dynamics and proximity networks of hub proteins at the prey-invading cell pole in a predatory bacterium
Source: J Bacteriol. 2024 Mar 12;206(4):e00014-24. doi: 10.1128/jb.00014-24 (PMC11025332; doi:10.1128/jb.00014-24)
Supplement: Supplemental figures — Figures S1 to S5. [file jb.00014-24-s0001.pdf]

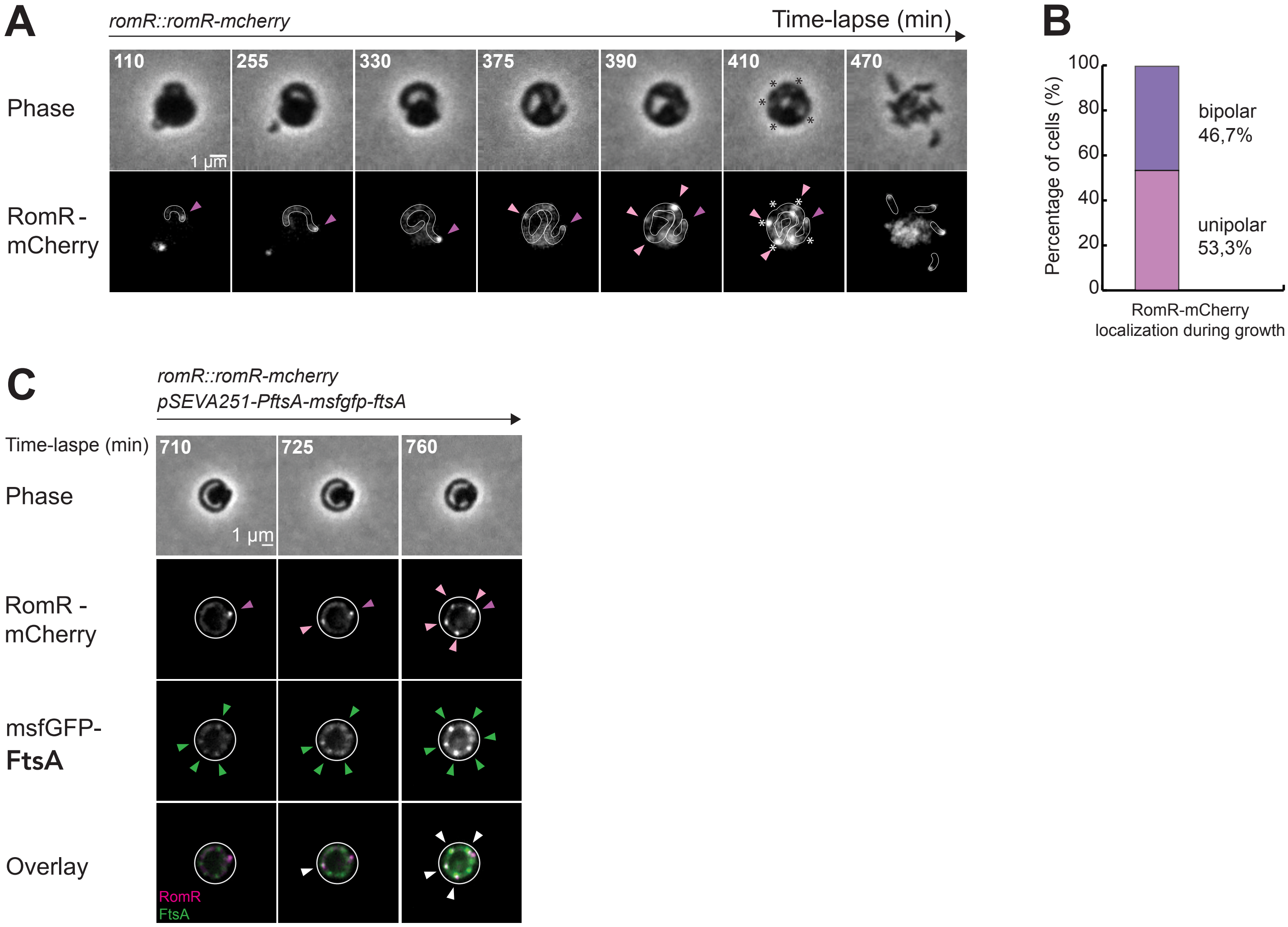
A
romR::romR-mcherry
Time-lapse (min)
110
255
330
375
390
410
470
Phase
1 µm
RomR - mCherry
B
Percentage of cells (%)
100
80
60
40
20
0
bipolar 46,7%
unipolar 53,3%
RomR-mCherry localization during growth
C
romR::romR-mcherry
pSEVA251-PftsA-msfgfp-ftsA
Time-laspe (min)
710
725
760
Phase
1 µm
RomR - mCherry
msfGFP-FtsA
Overlay
RomR
FtsA

# - Supp FIG 1 -

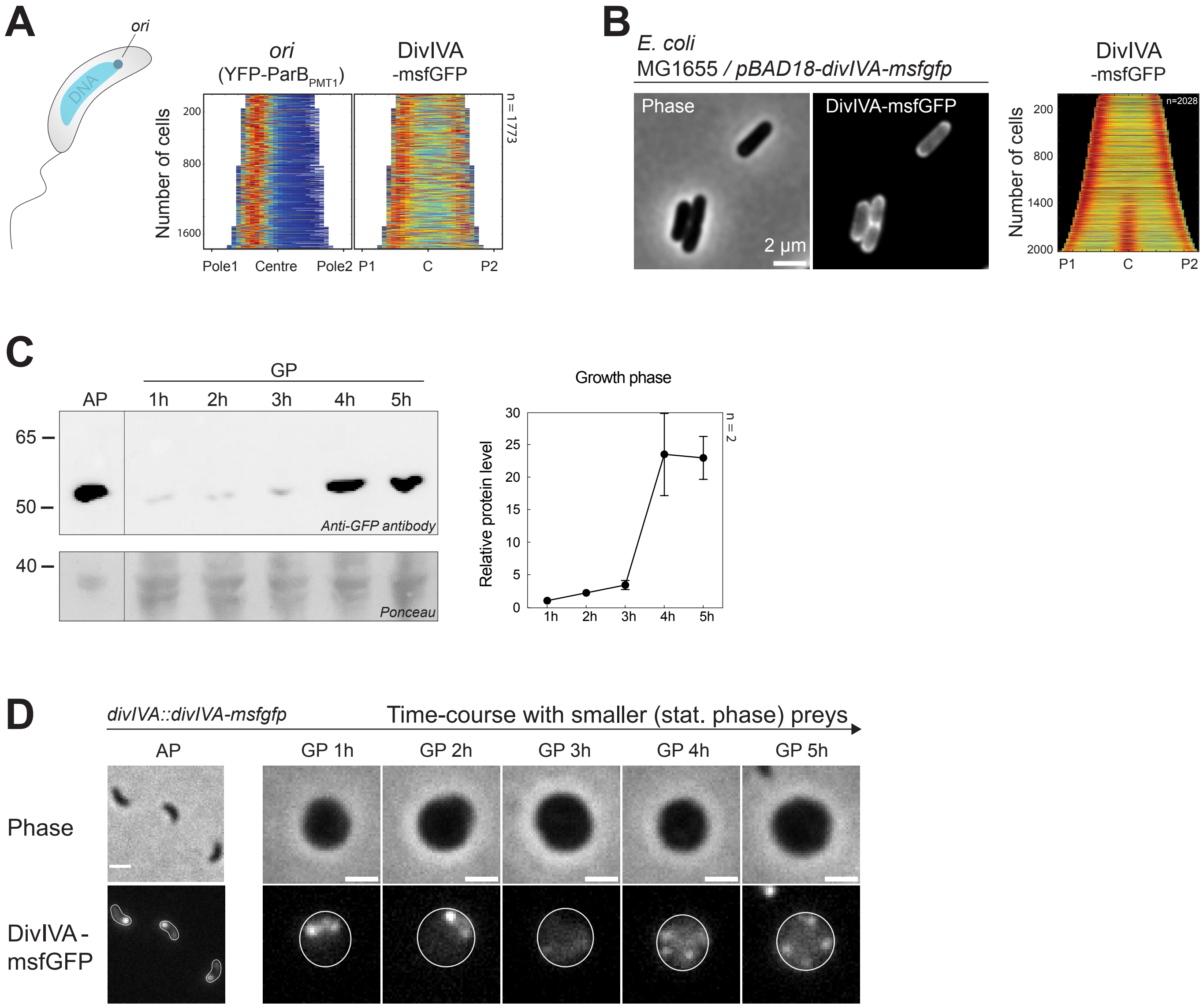

A
ori
DNA
ori
(YFP-ParB$_{PMT1}$)
DivIVA
-msfGFP
Number of cells
200
800
1600
n = 1773
Pole1
Centre
Pole2
P1
C
P2
B
E. coli
MG1655 / pBAD18-divIVA-msfgfp
Phase
DivIVA-msfGFP
2 µm
DivIVA
-msfGFP
n=2028
Number of cells
200
800
1400
2000
P1
C
P2
C
GP
AP
1h
2h
3h
4h
5h
65
50
40
Anti-GFP antibody
Ponceau
Growth phase
Relative protein level
30
25
20
15
10
5
0
n = 2
1h
2h
3h
4h
5h
D
divIVA::divIVA-msfgfp
Time-course with smaller (stat. phase) preys
AP
GP 1h
GP 2h
GP 3h
GP 4h
GP 5h
Phase
DivIVA-msfGFP

- Supp FIG 2 -

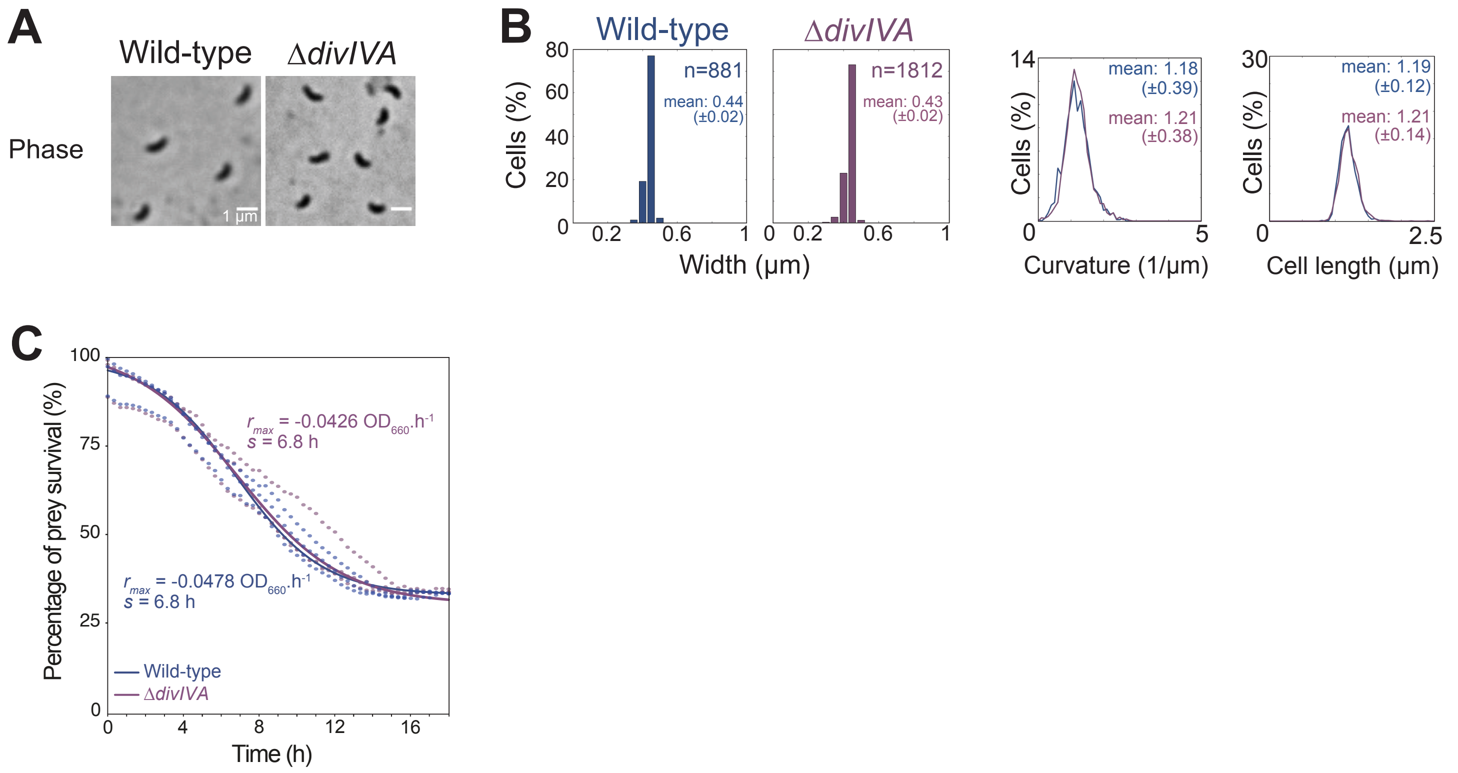
A
Wild-type
ΔdivIVA
Phase
1 µm
B
Wild-type
ΔdivIVA
Cells (%)
n=881
mean: 0.44
(±0.02)
n=1812
mean: 0.43
(±0.02)
Width (µm)
Curvature (1/µm)
mean: 1.18
(±0.39)
mean: 1.21
(±0.38)
Cell length (µm)
mean: 1.19
(±0.12)
mean: 1.21
(±0.14)
C
Percentage of prey survival (%)
r max = -0.0426 OD660.h-1
s = 6.8 h
r max = -0.0478 OD660.h-1
s = 6.8 h
Wild-type
ΔdivIVA
Time (h)

- Supp FIG 3 -

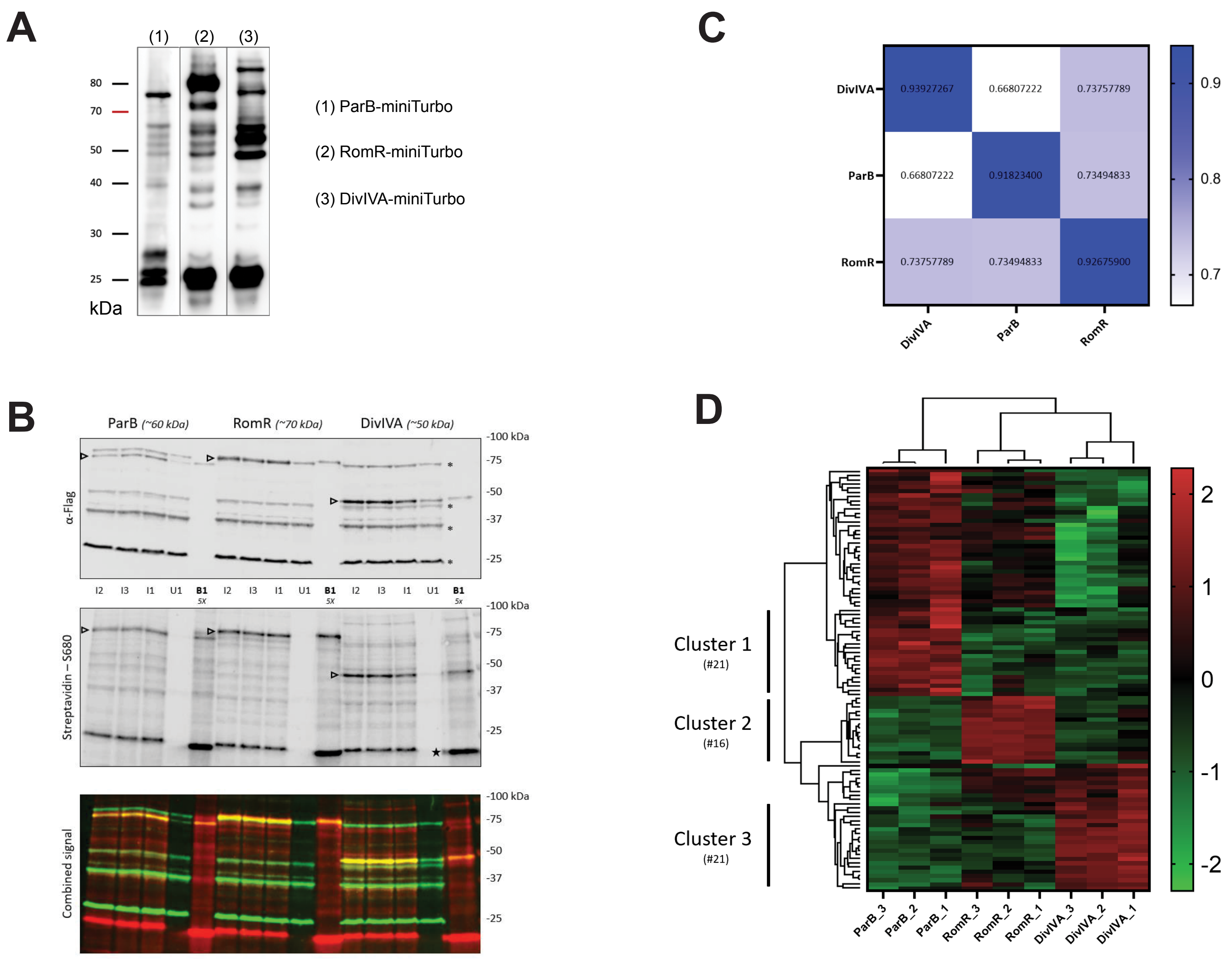
A
(1)
(2)
(3)
80
70
50
40
30
25
kDa
(1) ParB-miniTurbo
(2) RomR-miniTurbo
(3) DivIVA-miniTurbo
C
DivIVA
ParB
RomR
0.93927267
0.66807222
0.73757789
0.66807222
0.91823400
0.73494833
0.73757789
0.73494833
0.92675900
0.9
0.8
0.7
B
ParB (~60 kDa)
RomR (~70 kDa)
DivIVA (~50 kDa)
α-Flag
Streptavidin – S680
Combined signal
I2 I3 I1 U1 B1 5X
-100 kDa
-75
-50
-37
-25
D
Cluster 1 (#21)
Cluster 2 (#16)
Cluster 3 (#21)
2
1
0
-1
-2
ParB_3 ParB_2 ParB_1 RomR_3 RomR_2 RomR_1 DivIVA_3 DivIVA_2 DivIVA_1

- Supp FIG 4 -

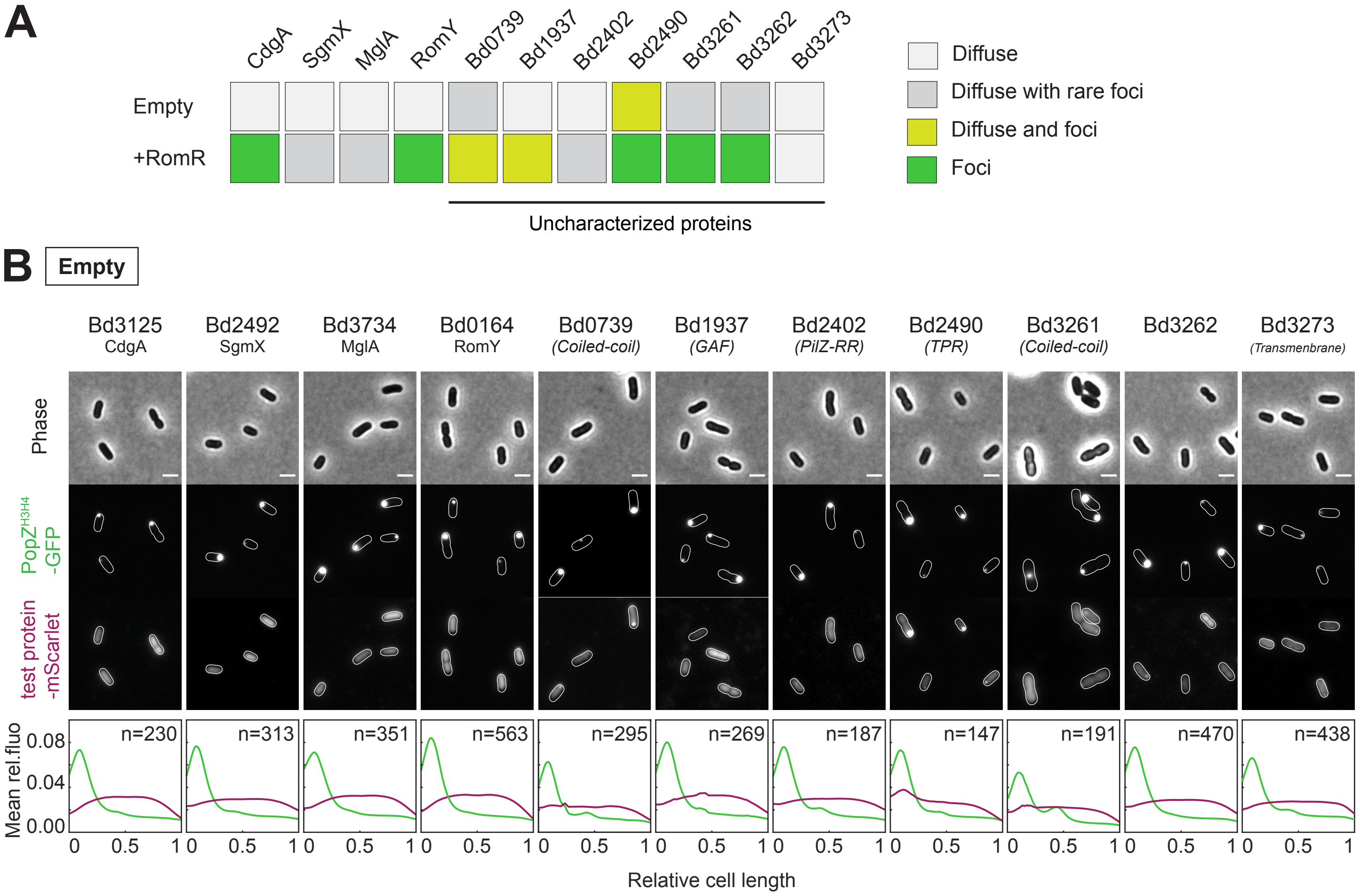

A
CdgA
SgmX
MglA
RomY
Bd0739
Bd1937
Bd2402
Bd2490
Bd3261
Bd3262
Bd3273
Empty
+RomR
Diffuse
Diffuse with rare foci
Diffuse and foci
Foci
Uncharacterized proteins
B
Empty
Bd3125 CdgA
Bd2492 SgmX
Bd3734 MglA
Bd0164 RomY
Bd0739 (Coiled-coil)
Bd1937 (GAF)
Bd2402 (PilZ-RR)
Bd2490 (TPR)
Bd3261 (Coiled-coil)
Bd3262
Bd3273 (Transmenbrane)
Phase
PopZ H3H4 -GFP
test protein -mScarlet
Mean rel.fluo
n=230
n=313
n=351
n=563
n=295
n=269
n=187
n=147
n=191
n=470
n=438
Relative cell length

# - Supp FIG 5 -

Legends for the supplementary figures

SUPP FIG 1 Characterization of the localization of RomR during the *B. bacteriovorus* growth phase. **(A)** Representative bdelloplast containing a GP *B. bacteriovorus* cell of the *romR::romR-mcherry* strain (GL1466) in which no second polar RomR-mCherry focus is visible during the growth of the filament. New foci appear along the cell body. The predators were mixed with exponentially grown MG1655 *E.coli* prey for 90 min prior time-lapse imaging with 5 min intervals. The phase contrast channel is on top, the mCherry channel at the bottom. Dark pink arrowheads point to the "old" RomR-mCherry focus at the invasive pole, light pink arrowheads to the new foci appearing during the growth phase, and asterisks to constriction sites. **(B)** Graphical representation of the percentage of cells with a unipolar (53,3%; as in A) or bipolar (46,7%, as in **Fig 1A**) localization of RomR-mCherry foci during growth (n=30). **(C)** Localization of RomR-mCherry compared to the early divisome component msfGFP-FtsA (GL2379). Representative bdelloplast containing a GP *B. bacteriovorus* cell (strain GL2379) mixed with exponentially grown GL522 *E. coli* prey for 90 min prior time-lapse imaging with 5 min intervals. From top to bottom, phase contrast, mCherry, GFP channels and overlay of both FP channels with RomR-mCherry in magenta and msfGFP-FtsA in green. The dark pink arrowhead points to the old RomR-mCherry focus, light pink arrowheads point to the new RomR-mCherry foci; green arrowheads to the msfGFP-FtsA foci; white arrowheads to the colocalized foci. Each time-lapse experiment was performed at least 3 times. *B. bacteriovorus* filament and bdelloplasts outlines were drawn manually based on the phase contrast images.

SUPP FIG 2 Characterization of the localization of DivIVA during the *B. bacteriovorus* attack and growth phases. **(A)** DivIVA-msfGFP localizes preferentially at the non-flagellated pole, visualized using the origin of replication on the chromosome (*ori*) via the *parS*$_{PMT1}$-YFP-ParB$_{PMT1}$ system previously used in *B. bacteriovorus* (8). Left: Schematic representation of an attack phase cell with the nucleoid in blue and the *ori* as a blue dot near the invasive pole. Right: Demographs of the fluorescent signals imaged in attack phase GL1636 cells (YFP-ParB $_{PMT1}$ on the left, DivIVA-msfGFP on the right), sorted by length and oriented with the YFP-ParB$_{PMT1}$ (*ori* marker) signal on the left, labelled as pole 1. Blue-to-red indicates low-to-high fluorescence intensities; the number of analysed cells is (n=)1773. **(B)** DivIVA$_{Bb}$-msfGFP uniformly localizes at both poles and septa when heterologously expressed in a *E. coli*. Left: Representative images of MG1655 *E. coli* strain expressing DivIVA-msfGFP from an inducible promoter on a plasmid (pBAD18). Phase channel on the left, GFP channel on the

right. Right: Demograph of the DivIVA-msfGFP fluorescent signal in *E. coli*, sorted by length, with random orientation. White-to-red indicates low-to-high intensities; the number of analyzed cells is (n=)2028. **(C)** DivIVA-msfGFP protein amount during the *B. bacteriovorus* cell cycle. A clear culture of AP cells for the *B. bacteriovorus divIVA::divIVA-msfgfp* strain (GL1620) was mixed with stationary phase MG1655 *E. coli* prey and samples from this predation mix were collected every hour and used for detection of DivIVA-msfGFP by immunoblot (C) or imaged (D). DivIVA-msfGFP protein amount increases at the end of the cell cycle. Left: whole-cell protein extracts from the time-course samples were used for immunoblotting with an anti-GFP antibody. Attack and growth phase samples were loaded on the same gel but only GP samples are compared since the loading material differs between the two phases. Molecular weight markers (kDa) are shown on the side. Ponceau staining (bottom) serves as a loading and transfer control and for normalization as done previously ((24) and see Methods). A representative replicate is shown. Right: Quantification of the relative protein level for each GP timepoint from two independent replicates. Protein levels, measured for each timepoint and replicate, were normalized by the corresponding Ponceau lane and the value of the first timepoint (see Methods). Plotted values are the mean relative levels from both replicates; error bars represent standard deviations. **(D)** Localization of DivIVA-msfGFP in *B. bacteriovorus* growing in smaller prey cells. Left: Representative phase contrast and GFP channel images of AP cells for the *B. bacteriovorus divIVA::divIVA-msfgfp* strain (GL1620). Right: Representative bdelloplasts containing a GP cell of the same strain imaged in time-course upon mixing with *E. coli* MG1655 as in C. Scale bars are 1 µm. *B. bacteriovorus* cells and bdelloplasts outlines were drawn manually based on the phase contrast images.

SUPP FIG 3 The absence of DivIVA does not impact the morphology of *B. bacteriovorus* (AP) cells. **(A)** Representative phase contrast images of the wild-type HD100 strain (GL734, left) and the HD100 Δ*divIVA* strain (GL1641, right). **(B)** Histograms of cell width, curvature, and cell length, comparing the AP cells of the wild-type and Δ*divIVA* strains. Mean values and graphical elements related to wild-type and Δ*divIVA* strains are coloured in blue and purple, respectively. The number of cells analysed is (n=) 881 for wild-type and 1812 for Δ*divIVA*. **(C)** Killing curves showing the percentage of prey survival over time when the *E. coli* prey are mixed with *B. bacteriovorus* wild-type (blue) or Δ*divIVA* (purple) strains. The maximum killing rate is given by the "r_{max}" values and the time when this maximum rate is obtained is given by the "s=" values. Mean curves obtained from three biological replicates are shown as plain or dotted lines, respectively. Values were normalized to the initial absorbance value.

SUPP FIG 4 Endogenous miniTurbo-based proximity labelling in *B. bacteriovorus*. (8) **(A)** Immunoblot using a Streptavidin-HRP conjugate on whole-cell protein extract to evaluate the distinct biotinylation pattern of each bait (fused to miniTurbo and expressed from their native locus). Biotin (50 μM) was added for 2h during the attack phase followed by 5h during the growth phase. Molecular weight markers (kDa) are shown on the side. Distinct biotinylation patterns indicate POI-specificity and *cis*- and *trans*-biotinylation activity of the protein fusions. **(B)** Immunoblots of fractions collected during the sample preparation for MS, demonstrating the efficient and specific capture of biotinylated protein material. Top: An anti-Flag antibody was used for the detection of the indicated bait protein fusions to the miniTurbo-Flag enzyme. Middle: A streptavidin/Alexa FluorTM 680 (S680) conjugate was used for the detection of biotinylated proteins. Bottom: Overlay of both immunoblots (anti-Flag in green and S680 in red). The overlay (yellow) signal shows that the bait fusion is biotinylated (as also pointed by the arrowheads on the two top blots). Unspecific signal (asterisks) and endogenously biotinylated protein (star) serve as loading controls. For each bait, the input samples of 3 replicates were analysed (I1 to 3), alongside the streptavidin unbound (U) and bound (B) fractions (5x concentrated) of the first replicate sample. **(C)** Heat map visualization of pairwise LFQ (label free quantification) correlations (Pearson), averaged per setup across the replicates (3 for each bait) analysed by MS following on-bead tryptic digestion, indicating a good correlation among the replicates while showing the variability between the replicates groups (i.e., the different baits). **(D)** Heat map representation of cluster analysis after ANOVA. The intensities of proteins with significantly different abundance ($p \leq 0.01$) in the respective proxisomes (total protein #100) are represented. Three clusters (clusters 1-3), respectively nearly exclusively enriched for RomR (Cluster 2), DivIVA (Cluster 3) and ParB (Cluster 1) putative interactors can be observed. Green-to-red indicates low-to-high intensities.

SUPP FIG 5 RomR directly interacts with several proteins of its proximity network. **(A)** Schematic representation of the POLAR assay results using RomR as a bait. The proteins used as prey are listed on top with their gene locus. The first row corresponds to the results obtained with the empty bait vector (shown in **B**), containing only PopZ^{H3H4}-GFP, while the second row corresponds to the results obtained with RomR as a bait (shown in **Fig 5**). Localization patterns observed for the mScarlet-tagged protein, with or without the RomR bait, are color-coded as shown on the right. We conclude on RomR-dependent recruitment, indicating direct interaction, for proteins in the light green (diffuse and foci) and dark green (foci) categories in the presence of RomR. **(B)** Representative microscopy images of the tested potential RomR partners tagged with mScarlet in the presence of the polarly localized PopZ^{H3H4}-GFP (encoded by the "empty" vector without bait). From top to bottom, the channels are phase contrast, GFP, mCherry. Scale bars are 2 µm. Cell outlines were obtained with Oufti (64). Graphs show the mean pole-to-pole profiles of relative fluorescence intensity in a population of cells, for the PopZ ^{H3H4}-GFP signal in green and the bait-mScarlet fusion in red; "n=" indicates the number of cells analyzed per condition.
